# Reprocessing and reuse of single-use medical devices in China: a pilot survey

**DOI:** 10.1186/s12889-019-6835-9

**Published:** 2019-04-30

**Authors:** Duojin Wang, Jing Wu

**Affiliations:** 1 Shanghai Engineering Research Center of Assistive Devices/School of Medical Instrument and Food Engineering, University of Shanghai for Science and Technology, Jungong Road 516, Shanghai, 200093 China; 2 School of Economics & Management, Tongji University, Siping Road 1500, Shanghai, 200092 China

**Keywords:** Single-use medical devices, Reprocessing, Reuse, National-wide survey

## Abstract

**Background:**

In China, reprocessing and reuse of single-use medical devices (SUDs) are banned. However, the actual situation has not been reported so far. The study aims to clarify the perceptions and concerns of various sectors of the community on the reuse of SUDs, and whether such practice exists. In addition, we are also wondering how acceptable the respondents are on this matter.

**Methods:**

A cross-sectional study based on a national survey which was conducted on the professional online questionnaire survey platform (www.wjx.cn) from July 26 to August 4, 2015. We analyzed the data according to the work fields, sex, age, education level, professional background and participants’ answers to 49 other questions.

**Results:**

Five hundred forty-four nationwide respondents belong to nine different work fields. In general, participants had positive attitudes towards the reprocessing and reuse of SUDs. However, many respondents doubted the hygienic and functional safety of the reprocessed SUDs. They also tended to think that the reuse of SUDs should have lower prices and more technical training as well as patient advocacy. Further analysis demonstrated the work fields, education level and professional background of respondents were statistically associated with their responses to certain questions.

**Conclusions:**

The research indicated that although the reuse of SUDs is prohibited legally in China, there were extensive reprocessing and reuse in hospitals. Most responses tended to accept reprocessed SUDs if safety and low prices were guaranteed. These existing contradictions and the lack of relevant research led to policy makers in China will confront numerous challenges in building and improving this use system of medical devices to meet escalating demands of social sectors.

**Electronic supplementary material:**

The online version of this article (10.1186/s12889-019-6835-9) contains supplementary material, which is available to authorized users.

## Background

With the development of newly fabrication materials such as plastic polymers the single-use medical devices (SUDs) have been booming since the late 1970s [1]. The original intention of SUDs stemmed from a desire to improve product performance and minimize the potential for disease transmission. Consequently, the SUDs are of great importance in modern medicine especially for minimally invasive technology in the past three decades. However, the increasing number of interventions and the consequent economic burden on health-care systems had led many countries to consider a reprocessing policy. Although there were controversial results associated with the safety and effectiveness of SUDs reprocessing and reuse [2–5], little evidence so far on its safety and efficacy has been published [6, 7], and the practice remains common in the most countries worldwide [1, 8–12].

Reprocessing of SUDs generally includes disassembling, decontamination, cleaning, inspection, testing, packing, relabeling, sterilization, and if necessary, refurbishing after they have been used on a patient for their intended purpose [13].

For decades now, the practice has been rationalized and legislated in many well-developed countries. In the United States, it is reported that more than 25% of hospitals reused at least one type of SUDs [7]. In August 2000, Food and Drug Administration (FDA) issued a policy on the reuse of SUDs. Currently, reprocessing of SUDs is regulated by FDA. Many hospitals delivered the used SUDs to third-party reprocessors instead of reprocessing reusable devices in-house. In 2008, nearly 70% of hospitals in the USA had agreements with third-party reprocessing companies [14]. At present, more than 100 different items are allowed to reprocess legally by FDA [15]. Currently Canada does not regulate the reprocessing of SUDs at the federal level due to the province to province variation. However, the regulatory background prohibits reprocessing and reuse of SUDs. The federal health care agency states that health care providers should not reuse SUDs unless the facility has established quality systems for reprocessing [8]. Even though this is common practice among hospitals and academic centers. It is reported that there are 28% of hospitals reprocessing and reusing SUDs, and 85% of which are reprocessed in-house [16, 17].

The European medical product market is currently worth around € 107 billion (est. 2011) and accounts for one third of the global market [18]. The number of SUDs is increasing because of safety concerns when SUDs are reprocessed [8]. Reuse of SUDs is undertaken in the most of European countries. According to the associated reports, there were more than 40% of German hospitals that used reprocessed SUDs [19], that was about 80% in Madrid, Spain [20], 37% in Denmark [21], approximately 10% in the United Kingdom (UK) and 100% in Norway [9]. The European Union (EU) does not have a uniform policy regarding to the reprocessing of SUDs. They differ in reprocessing activities depending on the regulations in individual Member States. For example, Germany has regulatory requirements: the German Act on Medical Devices and the Medical Device Operators Ordinance regulates the reprocessed details of SUDs, Robert Koch Institute (RKI) gives corresponding recommendation and the Federal Institute for Drugs and Medical Products (BfArM) for the reprocessing of devices [22]. In the Nordic countries such as Denmark, Finland and Sweden, similar regulatory frameworks have been adopted [23], but they are self-governed and need reviewing [24]. France has an absolute ban on reprocessing and reuse of SUDs and classifies reuse as deception of patients [8, 9]. In the UK the National Health Service does not allow the reuse of devices marked as single-use by manufacturers and there is a statement against the practice issued by the UK’s Medical Devices Agency [8, 9, 11]. However, so far in nearly all European countries reprocessing and reuse of SUDs may be undertaken and mostly without quality standards [8].

Compared to North America and Europe, the reprocessing of SUDs is relatively frequent in Japan. Two nation-wide surveys by Koh and Kawahara showed that the reuse rate was still at 86.2% in Japanese although the number of hospitals had decreased significantly from 2000 to 2003 [25]. This was attributed to the lack of appropriate regulatory system for surveillance [25]. The South Korea regulations do not allow recycling any medical waste component that include the most of SUDs. However, the hospitals refurbish the SUDs very commonly [26].

Many developing and transitional countries reuse even cheap SUDs very commonly like as needles, syringes and surgical gloves because of insufficient knowledge of healthcare workers, long-standing false beliefs of patients [27–29] and limited resources including facilities and finance [30]. In these countries, the reprocessing and reuse of SUDs are prevalent in an unregulated form due to their different economic scenario [9].

Brazil permits reprocessing of SUDs to use for many intervention procedures, however, the lack of basic guidance on how to carry out the practice is urgently in need [31]. Similarly, in India the reuse of SUDs continues in an unregulated manner due to a paucity of guidance from relevant government departments [9].

China is the most populous nation and second largest economy in the world. The China Food and Drug Administration (CFDA) has banned the reuse of SUDs [32]. As yet, there has been only a few studies on reuse of SUDs in China from the perspective of policy [33]. The lack of survey research of SUDs in Chinese health service leads to the shortage of relevant objective data, and this further might result in many problems concerning health regulation formulating, medical education promoting and establishment of medical service quality system.

Considering above circumstances, we have conducted a national-wide pilot survey of reprocessing and reuses of SUDs mainly for the purpose to clarify the current perceptions and concerns of the social sectors. In addition, we are also wondering whether such practice exists and how acceptable for the respondents. These investigations may be helpful to set up the appropriate regulatory and educational system of the policy for SUDs in China.

## Methods

Our study is a cross-sectional analysis of a national-wide survey which was carried out on the professional online questionnaire survey platform (www.sojump.com) from July 26, 2015 to August 4, 2015. This survey focused on the general population, but had some directional features, in other words, we aimed to include both medical device-related industry participants and the general public. Therefore, the link of the questionnaire was randomly sent to the staffs in different industries, and then they promoted it through the communication software. If someone is interested in the topic, open the link on the phone to fill it out directly. The logical relationship between the questions in the questionnaire has been defined before sending. If the respondents have logic errors or miss out questions in the process of filling in, they will be prompted and cannot submit. The successful submission will be regarded as a valid questionnaire. The selection bias due to non-entirely random participation will most likely result in significant uneven industry distribution of respondents, therefore we eliminate this part of data to minimize the bias in order to reduce the interference of subgroups with too few samples. Finally, 544 valid questionnaires were received.

Considering the sensitivity of the issue and the reliability of responses, sharp questions were avoided in this survey. The questionnaire included questions in relation to the characteristics of the participants, basic position and awareness about single use and reuse of medical devices, the cleaning, sterilization and other technical problems of medical devices in both use modes. In addition, the questions were aimed to collect information on the ethics issue, insurance payment and corresponding regulatory actions. The questionnaire consisted of 49 closed-ended questions with responses measured using a 7-point Likert scale except the basic demographic information of respondents. There are two purposes for using the 7-point scale. Firstly, the responses can be classified in more detail; more importantly, the survey requires people to answer multiple closed-ended questions online. Secondly, compared with the 3-point or 5-point scale, the 7-point scale can make the respondents spend more time to think and answer, so the results can more accurately reflect the respondent’s own real thoughts. In the process of analysis, we regarded “strongly disagree”, “disagree” and “somewhat disagree” in the 7-point as disagreement, that is, negative choice. “Somewhat agree”, “agree” and “strongly agree” are regarded as positive choice. (The questionnaire can be found in Additional file 1 for reviews but is not intended to be part of the manuscript. In order to make the related description easier to understand, the serial number of every question will be added in corresponding contents).

We used frequencies, percentages, and graphical display for descriptive analysis. We applied the Chi-square test to measure differences in respondents’ choice of all questions in various work fields, education levels and professional backgrounds. In addition, correlation analysis and multinomial logistic regression was conducted to further clarify whether and how respondents’ personal information influenced their choices. We reported crude odds ratios (ORs) and 95% CIs. Statistical data were entered into a database and processed using SPSS software (Version 20). *P*-Value less than 0.05 was considered as significant.

## Results

**Fig. 1:**
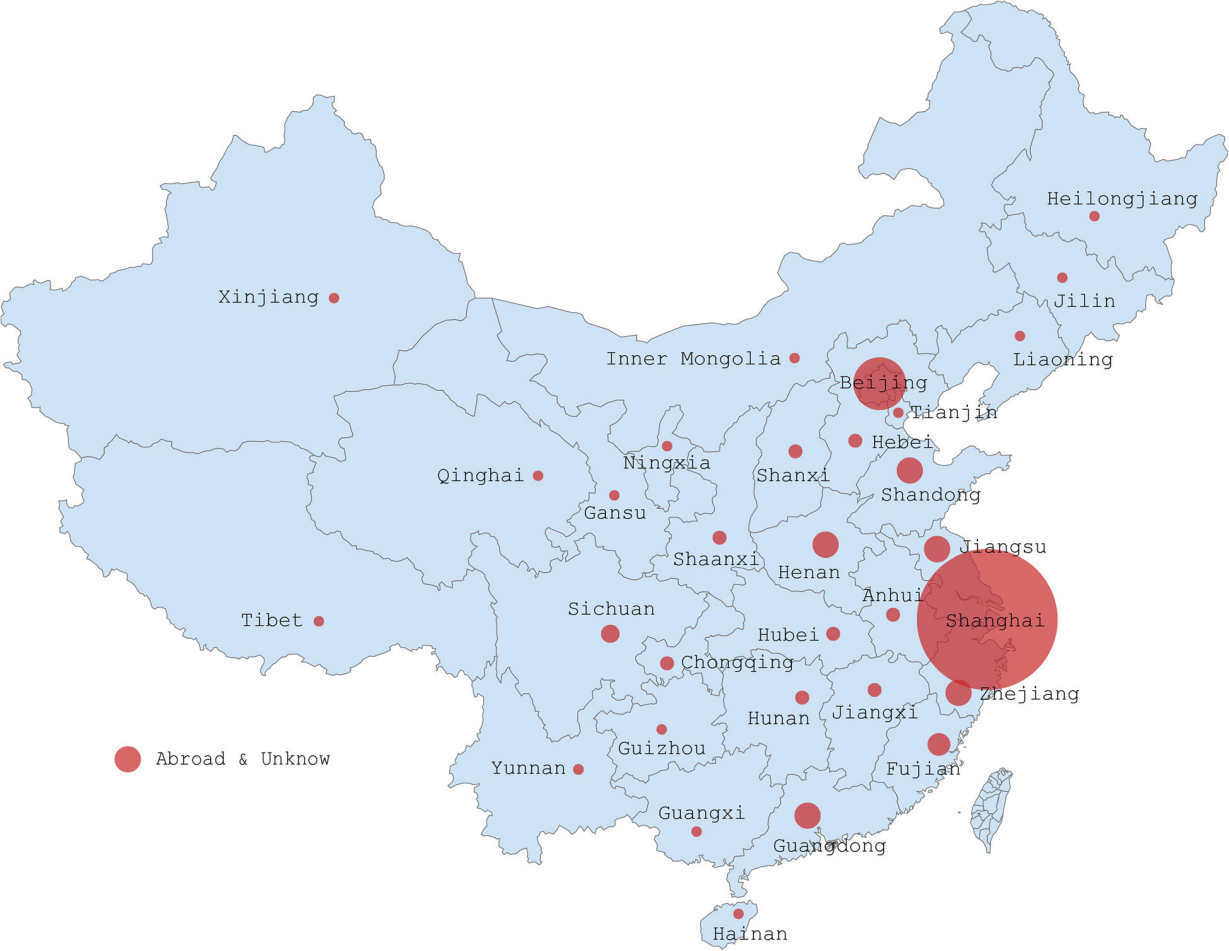
Distribution of responses. Authors’ drawing based on survey data. The vector Map of China is quoted from the open access archive of HIGHCHARTS, https://code.highcharts.com/mapdata/ (accessed 4 April 2018). Based on this background, we further mapped the distribution of samples in different regions. The authors acknowledge the source. Our survey was conducted online. The distribution is based on the IP data of the submission. In the figure, the size of red dot represents the number of respondents in the corresponding region

The basic information of the sample is shown as Table 1, 544 respondents from 31 provinces, autonomous regions, municipalities or other regions (Fig. 1 shows detailed distribution) belong to nine different work fields, and, of these, medical device manufacturers (34.8%), staff members in the hospital (32.2%) and regulatory authority of medical devices (13.2%) made up the majority (A1). The number of female (54.6%) in the survey was slightly larger than male (45.4%) (A2). The age group shows a distinctly uneven distribution. 26 to 35-year-old respondents accounted for around 40%, 36 to 60-year-old for 53%, and others only 7% (A3). 492 of the 544 (more than 90%) respondents have bachelor degrees or above (A4). Furthermore, Table 1 also indicates that most people have received medical related professional education such as clinical medicine (28.7%), pharmacy (18.6%) and relevant majors of medical device (24.3%) (A5).

**Table 1:** Basic information of the respondents in the survey

Sample Info.	Classification	No. of sample (*n* = 544)	Percentage (95% CI)
Work fields	Medical device manufacturers (foreign enterprise/ joint venture)	80	14.71% (11.73%~ 17.68%)
	Medical device manufacturers (state enterprise/private enterprise)	109	20.04% (16.67%~ 23.40%)
	Managing agent of medical device	32	5.88% (3.91%~ 7.86%)
	Clinician/Nurse	71	13.05% (10.22%~ 15.88%)
	Other staff member in the hospital	104	19.12% (15.81%~ 22.42%)
	Health insurer	1	0.18% (0~0.54%)
	Regulatory authority of medical devices	72	13.24% (10.39%~ 16.08%)
	Health regulator (infection control)	8	1.47% (0.46%~ 2.48%)
	Patient and other staff	67	12.32% (9.55%~ 15.08%)
Gender	Male	247	45.40% (41.22%~ 49.59%)
	Female	297	54.60% (50.41%~ 58.78%)
Age group	Under 25 years old	27	4.96% (3.14%~ 6.79%)
	26~35 years old	218	40.07% (35.96%~ 44.19%)
	36~45 years old	179	32.90% (28.96%~ 36.85%)
	46~ 60 years old	111	20.40% (17.02%~ 23.79%)
	Above 60 years old	9	1.65% (0.58%~ 2.73%)
Education level	Master or above	185	34.01% (30.03%~ 37.99%)
	Bachelor	307	56.43% (52.27%~ 60.60%)
	Other degree	52	9.56% (7.09%~ 12.03%)
Professional background	Clinical medicine	156	28.68% (24.88%~ 32.48%)
	Pharmacy	101	18.57% (15.30%~ 21.83%)
	Relevant majors of medical device	132	24.26% (20.66%~ 27.87%)
	Relevant majors of economics/ management/ literature/ law	36	6.62% (4.3%~ 8.71%)
	Other professional backgrounds	119	21.88% (18.40%~ 25.35%)
Sum		544	100%

### The basic position and awareness on single use and reuse of medical devices

**Fig. 2:**
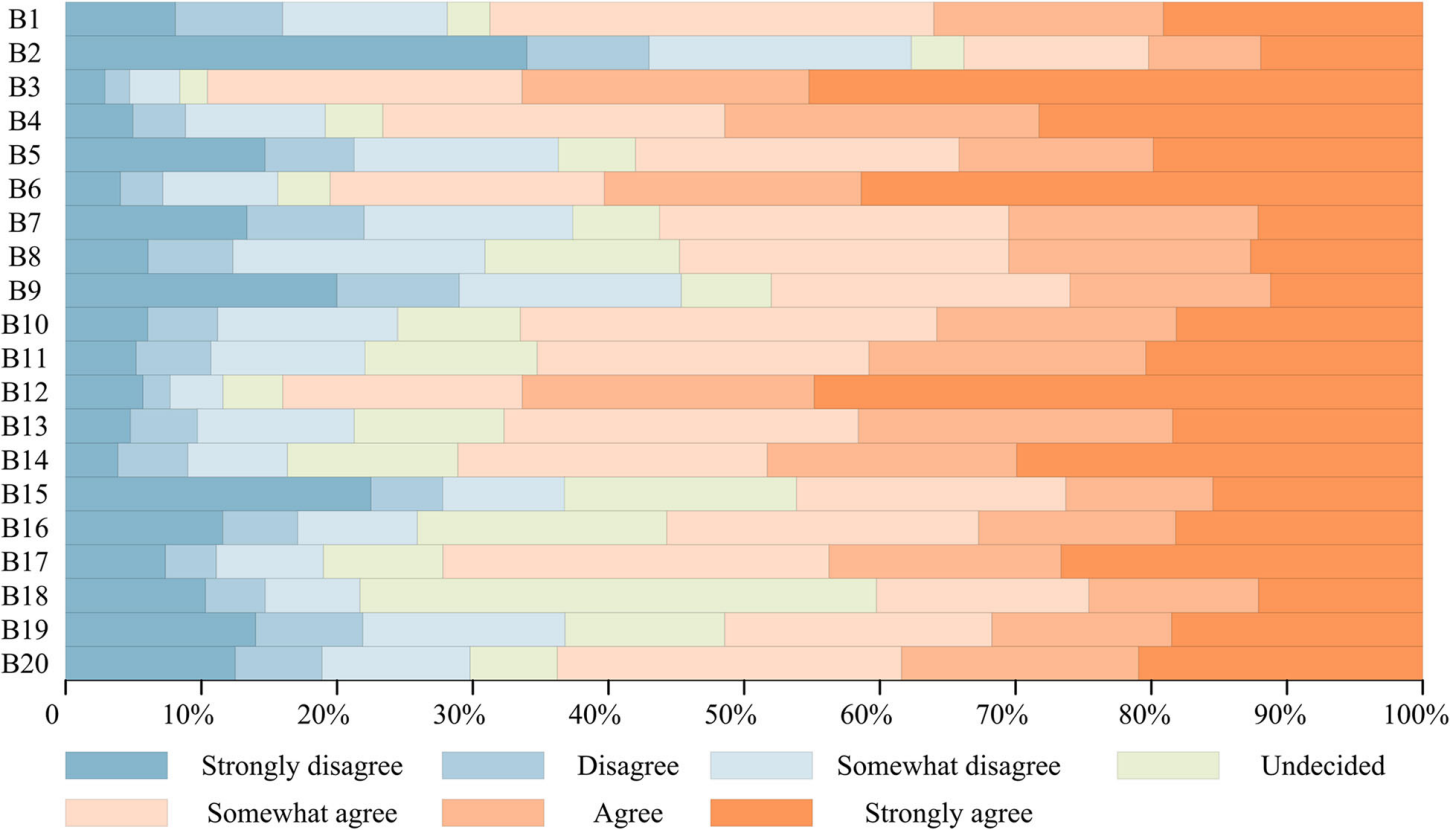
Graphical percentage display of questions in part B of the questionnaire. Authors’ drawing based on statistical data of questions in part B of the questionnaire. In the description, “Strongly disagree”, “Disagree” and “Somewhat disagree” were regarded as negative choice. “Somewhat agree”, “Agree” and “Atrongly agree” are regarded as positive choice

**Fig. 3:**
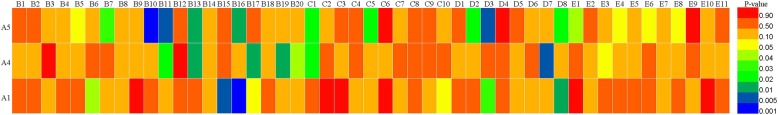
Heatmap of correlation analysis between respondents’ personal information and their responses. The respondents’ gender and age belong to their natural attributes, which have trifling influence on their choice and have no significance for further analysis. Therefore, we only analyze the correlation between the respondents’ work fields, education level, professional background and their answers. *P*-Value less than 0.05 was considered as significant correlation

As shown in Fig. 2, in general, participants had positive attitudes towards the reprocessing and reuse of SUDs. For example, 68.8% of respondents agreed that designing and producing more reusable medical devices can better reflect the concept of reducing waste and recycling economy (B1). A great majority of the respondents believed that medical devices are used as disposables for health security (89.5%) (B3) and safe of use (76.7%) (B4). Compared with the reusable medical devices, they (80.5%) tended to deem that health security of SUDs is higher from the perspective of infection control (B6), and the work field (“health insurer”) had a great influence on the answer (*P* = 0.039) (Fig. 3). However, there are plenty of doubters (45.2%) who pointed out that the safety of the reprocessing and reuse of SUDs cannot be guaranteed even through strict and regulated management (B9). When asked if they have ever seen the reuse of SUDs or ever reused SUDs, 200 of 544 (36.8%) indicated that they have not, 17.1% undecided, and 46.1% admitted (B15). Regarding this issue the differences are statistically significant by work field (*P* < 0.01) (Fig. 3). Among all work field groups, the “patient and other staff” were the last one who agree with this statement. Moreover, only 55.7% of respondents thought that the SUDs were being reused in hospitals (B16). The analysis results revealed that there was significant correlation of this question with work field (*P* < 0. 01) (Fig. 3), in which “other staff member in the hospital” showed obvious disagreement. Probably the respondents did not understand the reuse situation of SUDs in other countries, 207 of 544 (38.1%) said they had no idea whether the reuse of SUDs are allowed in some countries (B18).

In addition, the analysis results indicated that there was statistic relationship between some questions and the education level of the respondents. As shown in Figs. 2 and 3, 355 of 544 (65.3%) considered that manufacturers would prefer to produce SUDs because of faster and easier profits (*P* = 0.022) (B11). However, the reprocessing of medical devices including recovery, cleaning, sterilization and reuse in house could be more effective and safer than in manufacturing facility (51.5%, *P* = 0.01) (B19). Most people (72.2%) did not know much about the reuse of high-value consumables and need more knowledge as well as details to make judgments (*P* < 0.01) (B17). But nonetheless, many of them (67.6%) still agreed that reusable medical devices are more cost-effective than SUDs (*P* < 0.01) (B13), they (63.8%) also supported the reuse of high-value consumables (*P* = 0.043) (B20).

### The cleaning, sterilization and other technical problems on single use and reuse of medical devices

**Fig. 4:**
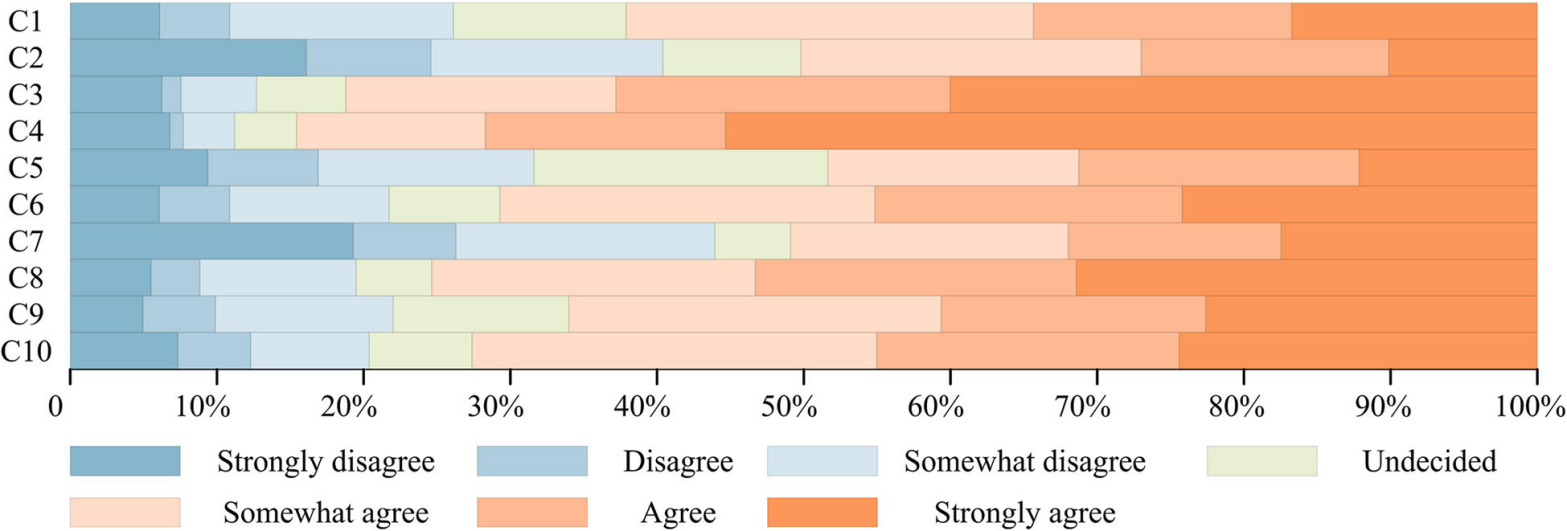
Graphical percentage display of questions in part C of the questionnaire. Authors’ drawing based on statistical data of questions in part C of the questionnaire

Figure 4 illustrated the graphical percentage of the respondents’ answers to the cleaning, sterilization and other technical problems on single use and reuse of medical devices. It can be seen that many respondents doubted the hygienic and functional safety of reprocessed SUDs. 220 of 544 (40.4%) believed that SUDs are considerably different from reusable ones in actual use, 50.2% denied (C2). There were 172 of 544 (31.6%) objections about whether existing cleaning and sterilization technologies can ensure the hygienic safety of the reprocessed medical devices, 20% were undecided, and approximately 50% agreed (C5). Although the most (84.6%) believed that reliable cleaning and sterilization technologies are required to ensure hygienic safety in reuse of SUDs (C4), 66% still had a negative attitude toward the reliability of reprocessing of SUDs psychologically (C9). 338 of 544 (62.1%) considered that manufacturers would prefer to produce SUDs because of difficult design and production of reusable medical devices (C1). The analysis results showed that there was significant correlation of this question to education level (*P* = 0. 025) (Fig. 3), among them, “Master or above” is more inclined to oppose. Anyway, people (72.6%) still agreed that the reuse of high-value consumables would reduce the treatment of medical waste (C10).

### The ethics issue and insurance payment on single use and reuse of medical devices

**Fig. 5:**
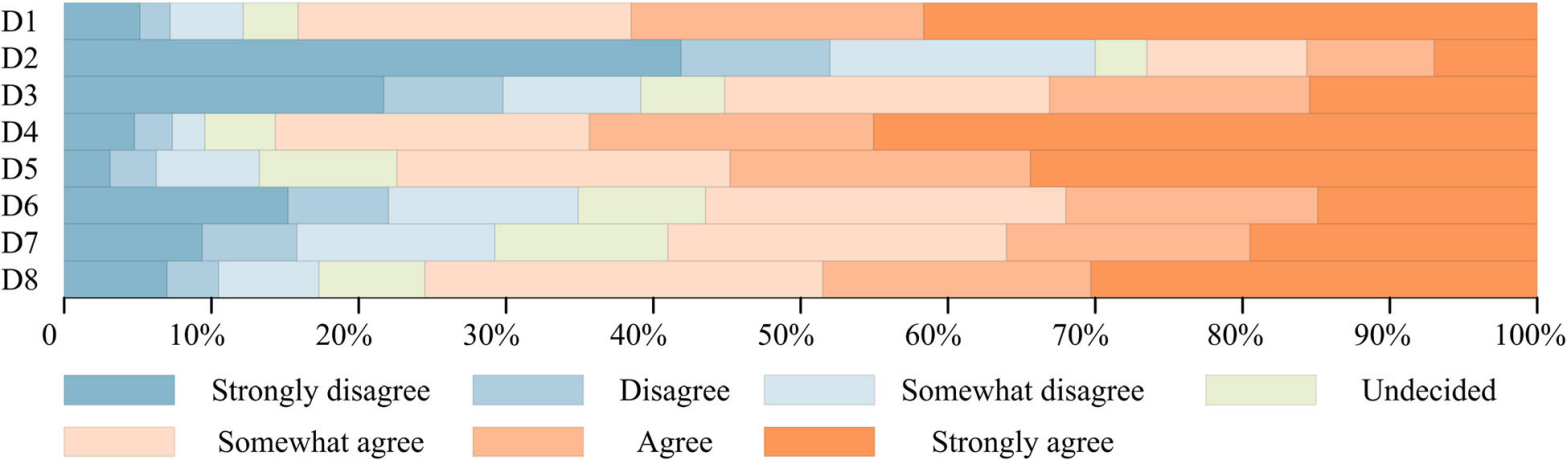
Graphical percentage display of questions in part D of the questionnaire Authors’ drawing based on statistical data of questions in part D of the questionnaire.

Based on the percentage shown in Fig. 5, it is widely believed that if SUDs are reused, prices should be lower (84.2% of respondents agreed) (D1) and more technical training and patient advocacy provided (85.7% of respondents agreed) (D4). Possibly considering the safety, people have different attitudes towards using reprocessed SUDs for themselves. 300 of 544 (55.1%) said that they did not mind under the premise of safety assurance, 39.2% still held the opposite attitude (D3). Further analysis result showed strongly disagreement to “Medical device manufacturers” and “Health regulator” (*P* = 0.033) (Fig. 3). However, 56.4% said that if the cost could be greatly reduced, then they were willing to accept the reuse of SUDs (D6). In addition, most responses (59.0%) indicated that reusable medical devices were certainly cheaper than SUDs, 11.8% undecided, and 29.2% held opposing attitudes. Further analysis indicated that this question was associated with education level (*P* < 0.01) (D7) (Fig. 3). Similarly, more respondents (75.6%) thought that a large number of SUDs would affect the environment (D8). This was also correlated to work field according to statistical analysis (*P* = 0.018) (Fig. 3).

### The supervision and management on single use and reuse of medical devices

**Fig. 6:**
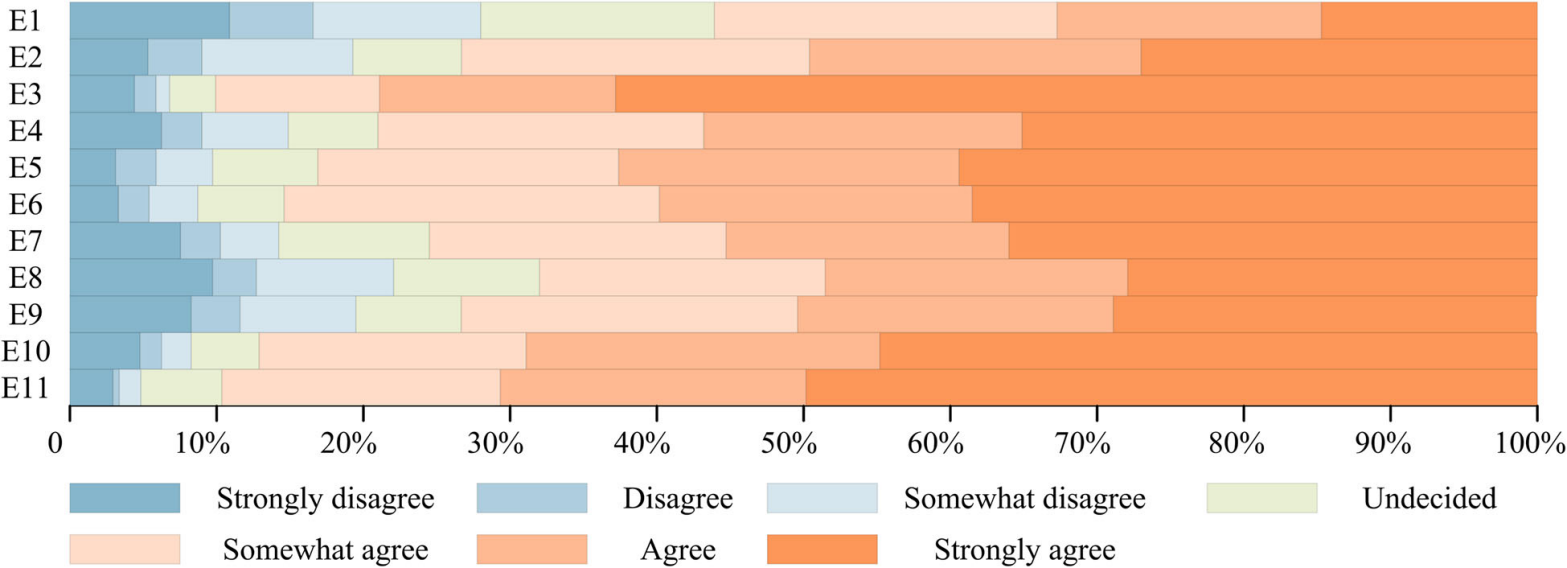
Graphical percentage display of questions in part E of the questionnaire. Authors’ drawing based on statistical data of questions in part E of the questionnaire

Please see Fig. 6 for respondents’ choice of questions in this section. People (89.7%) generally thought that the relevant policy regarding reuse of SUDs is not enough in China currently (E11). If the SUDs are reused after treatment, 490 of 544 (90.1%) believed that it should have a special management system (E3), 87.1% deemed that it should be determined by the professional departments to meet the relevant standards (E10), central sterile supply department (CSSD) should meet the standard of high-level hospital (75.4% held this view) (E7). In addition, there were 370 respondents (68%) who believed that professional services should be in charge of the sterilization (E8). Relative to the reusable medical devices, 73.3% thought that SUDs were safer from the health perspective (E2), 79% considered that the risk of SUDs’ use was easier to control (E4), and 83.1% held the opinion that the use of SUDs was easier to tracing and traceability (E5). 465 of 544 (85.5%) agreed that the SUDs could be managed effectively if there was a complete traceability management system (E6). With respect to how to determine whether medical devices should be reused, 398 respondents (73.2%) said that it was more reasonable from clinical needs rather than the regulatory authorities (E9). Nevertheless, there was still disagreement on some supervision measures, e.g. 355 of 544 (65.3%) thought the medical devices that labeled as single-use were easier through the process of marketing approval than labeled as reusable, 16% undecided, and approximately 28% denied (E1).

### Further analysis of key data

Figure 3 shows several indicators that respondents’ personal information was statistically associated with their responses to certain questions. Considering the number and importance, we selected some key issues (B10, B13, B15, C5, D3 and D7) and used regression analysis to further examine correlations between these personalities and their choices.

In the multinomial logistic regression, which estimated the influence of people’s choices on the dependent variable of several selected questions, the independent variables for respondents’ work fields, education level, professional background were included respectively (Table 2).

**Table 2:** Odds ratios (ORs) for respondents’ choice of selected questions by work fields, education level and professional background respectively

Personal information		Agreement (Crude OR, 95% CI)^a^					Disagreement (Crude OR, 95% CI)^a^	
Independent variable		B10	B13	B15	C5	D3	B13	D7
Work fields	Patient and other staff			1.00				
	Medical device manufacturers (foreign enterprise/joint venture)	–	–	3.71 (1.64–8.40)	–	–	–	–
	Managing agent of medical device	–	–	3.80 (1.42–10.18)	–	–	–	–
	Clinician/Nurse	–	–	3.74 (1.68–8.32)	–	–	–	–
Education level	Other degree	–	–	–	–	–	1.00	1.00
	Master or above						2.65 (1.11–6.32)	2.36 (1.06–5.22)
Professional background	Other professional backgrounds	1.00	1.00		1.00	1.00		
	Clinical medicine	2.31 (1.29–4.14)	2.14 (1.14–4.01)	–	2.03 (1.15–3.59)	2.14 (1.29–3.55)	–	–

^a^ The OR value given means that *p* < 0.05

-indicates that there is no statistically significant correlation between the personal information of respondents and their choices for the selected questions

For question “B15- I have seen the reuse of SUDs/ I have reused SUDs”, 46.1% of the responses were positive, and all of which were more inclined to agree with the statement except health insurer and health regulator (the sample size of these two work fields was too small to be statistically significant). Compared with the general patients, the number of medical device manufacturers (foreign enterprise/joint venture), managing agent of medical device and clinician/nurse who have reused or seen the reuse of SUDs was more than 3.5 times (OR > 3.5).

In addition, the higher the respondents’ academic qualifications, the more tended they are to hold opposite or uncertain attitudes, for instance, people with Master degree or above had negative responses toward the issue that “SUDs are more expensive” compared with those with none (OR = 2.65 for B13 and 2.36 for D7). They also did not believe that it could be more effective and safer to reprocess the SUDs in house (OR = 2.66 for B19).

Different professional backgrounds lead to different choices. In general, clinical medicine-related respondents are more professional on some issues. In contrast to others (the fifth category in A5--not pharmacy, medical device and economics/ management/ literature/ law related majors), they would prefer to agree that reuse of medical devices was beneficial to lower medical costs (OR = 2.31 for B10) and reusable medical devices were more cost-effective than SUDs (OR = 2.14 for B13). They were also more inclined to believe that existing technologies could ensure the safety of processed medical devices (OR = 2.03 for C5). They did not mind using reprocessed SUDs if safety is guaranteed (OR = 2.14 for D3).

## Discussion

Reprocessing and reuse of SUDs are undertaken in most of countries worldwide. Although the CFDA has banned them, the actual situation has not been reported at present. Our nationwide survey aims to clarify the perceptions and concerns of various sectors of the community on the reuse of SUDs, and whether such activity exists in practice. In addition, we are also wondering how acceptable the respondents are on this matter.

From the statistical results of the questionnaire, it is known that the questions related to whether reuse of SUDs exists (B15 and B16) have significant statistical correlation to the respondents’ work fields. In all categories, hospital staff (clinician/nurse and other staff member in the hospital) and medical device manufacturers are direct stakeholders associated with this issue, the credibility of their responses is therefore relatively high. Further analysis shows that 46.1% of respondents experienced the reuse of SUDs, in which hospital staff accounted for 35.5%, medical device manufacturers 35.9%. In addition, 55.7% of respondents believe that SUDs are being reused in hospitals, where hospital staff and medical device manufacturers reached 27.7 and 40.6% respectively. These data seem to demonstrate that the phenomenon has always existed to some extent. To test this hypothesis, we conducted dozens of on-the-spot visits and found out that the practice actually exists in almost every hospital. However, owning to the ban of reuse of SUDs by law, many respondents were likely to be avoiding or denying this topic deliberately, this is probably because some respondents such as health regulator may not wish to face up to this fact.

In China, public health insurance does not cover all medical services currently yet, in particular, a majority of high-value consumables must be paid by the patients themselves. Therefore, it is not difficult to understand the patient’s acceptance of reuse of SUDs and the resulting low cost in the responses. Meanwhile, widespread denouncements have urged the Chinese government to improve medical pricing scheme continuously, e.g. policy makers have set relatively low prices for these consumables. In such situation, however, hospitals have to depend on reuse of SUDs simply to remain open. Reuse of SUDs with the risk of violation of laws, the most crucial reason is the financial burden to the hospitals. Moreover, from the perspectives of economy, environmental protection, safety, and management, everyone seems to have a general acceptable attitude towards the reuse of SUDs, and they believe that current policy and patient publicity is not yet in place. Fortunately, the Chinese government has been seeking the right solution, and, we also learned from the reports of the two sessions (the National People’s Congress and the Chinese people’s political consultative conference) this year, the Chinese government will start on large-scale institutional reforms to solve these problems.

Research on the safety of the reuse of SUDs is relatively lacking in China, because conducting related studies are considered insignificant when there is prohibition in laws. It also reflects that the prohibition on the reuse of SUDs is not supported by any scientific research.

Our research has inevitably some limitations. There are only few samples in some work field, e.g. the responses of health insurer and health regulator, so the data collected may have been subjected to career bias. Given the complexity and sensitive nature of the topic, it was difficult for a single respondent to answer all questions accurately. The survey also did not include specific region, medical institutions, types and frequency of reuse, and did not involve detailed reprocessing mode. These issues are expected to be carried out in our future studies.

## Conclusion

This research demonstrated that there are extensive reprocessing and reuse of SUDs under the circumstance of prohibition by law in China. Most respondents indicated that they could accept reprocessed SUDs if safety and low prices were guaranteed. These facts also illustrate that the existing relevant policy has some degree of irrationality. China’s policy makers understand that simple bans have no practical effect, and gradually a rational use system of SUDs will be developed to meet escalating demands of social sectors. The lack of research on the reuse of SUDs pushes policy makers in China to confront numerous challenges including conflicting policy interests, a weak regulatory framework, to be improved educational mechanism, and lack of enforcement capacity for building and improving this use system of medical devices.

Reuse of SUDs is banned in China currently, however with so many challenges, government is very likely allowing some SUDs such as high-value consumables to be reused gradually under appropriate supervision. This is bumpy undoubtedly, but nevertheless, there are already many successful cases worldwide.

## Additional file

Additional file 1: Questionnaire on reprocessing and reuses of SUDs in China. This data is the questionnaire involved in this study. The questionnaire consisted of 49 closed-ended questions with responses measured using a 7-point Likert scale except the basic demographic information of respondents. (XLSX 13 kb)

## Abbreviations

BfArM: Federal Institute for Drugs and Medical Products
CFDA: China Food and Drug Administration
EU: European Union
FDA: Food and Drug Administration
ORs: Odds Ratios
RKI: Robert Koch Institute
SUD: Single-use medical devices
UK: United Kingdom

## References

[1] Tessarolo F, Caola I, Nollo G, Komorowska MA, Olsztynska-Janus S (2011). Critical issues in reprocessing single-use medical devices for interventional cardiology. Biomedical engineering, trends, research and technologies.

[2] Luijt DS, Schirm J, Savelkoul PHM, Hoekstra A (2001). Risk of infection by reprocessed and resterilized virus-contaminated catheters: an in-vitro study. Eur Heart J.

[3] Port FK, Wolfe RA, Hulbert-Shearon TE, Daugirdas JT, Agodoa LYC, Jones C, Orzol SM, Held PJ (2001). Mortality risk by hemodialyzer reuse practice and dialyzer membrane characteristics: results from the USRDS dialysis morbidity and mortality study. Am J Kidney Dis.

[4] Hakansson MA (2014). Reuse versus single-use catheters for intermittent catheterization: what is safe and preferred? Review of current status. Spinal Cord.

[5] Day P. What is the evidence on the safety and effectiveness of the reuse of medical devices labelled as single-use only? Christchurch: New Zealand Health Technology Assessment (NZHTA); 2004:53.

[6] United States Government Accountability Office. Report to the Committee on Oversight and Government Reform, House of Representatives. Reprocessed single-use medical devices. FDA oversight has increased, and available information does not indicate that use presents an elevated health risk. 2008. GAO-08-147.

[7] Kwakye G, Pronovost PJ, Makary MA (2010). Commentary: a call to go green in health care by reprocessing medical equipment. Acad Med.

[8] Popp W, Rasslan O, Unahalekhaka A, Brenner P, Fischnaller E, Fathy M, Goldman C, Gillespie E (2010). What is the use? An international look at reuse of single-use medical devices. Int J Hyg Envir Heal.

[9] Hussain M, Balsara KP, Nagral S (2012). Reuse of single-use devices: looking back, looking forward. Natl Med J India.

[10] Tessarolo F, Disertori M, Guarrera GM, Capri S, Nollo G (2009). Reprocessing single-use cardiac catheters for interventional cardiology. A cost-minimization model for estimating potential saving at departmental scale and national level. Italian Journal of Public Health.

[11] Alfa MJ, Castillo J (2004). Impact of FDA policy change on the reuse of single-use medical devices in Michigan hospitals. Am J Infect Control.

[12] Noble M. Interventions to allow the reuse of single-use devices: Brief review. In: prepared by RAND Corporation, University of California, Johns Hopkins University & ECRI Institute. Making Health Care Safer II: An Updated Critical Analysis of the Evidence for Patient Safety Practices. Evidence Report/Technology Assessment, No. 211; 2013. 117–121. Available at https://www.ncbi.nlm.nih.gov/books/NBK133363/. (Accessed 27 June 2017).

[13] U.S. Department of Health and Human Services, Food and Drug Administration, Center for Devices and Radiological Health. Guidance for Industry and for FDA Staff: Enforcement Priorities for Single-Use Devices Reprocessed by Third Parties and Hospitals. 2000, Available at https://www.fda.gov/downloads/MedicalDevices/DeviceRegulationandGuidance/GuidanceDocuments/ucm107172.pdf. (Accessed 27 June 2017).

[14] Moduga A. Reduce, reuse, recycle: reprocessing medical devices. 2010. Available at http://www.hospitalmanagement.net/features/feature80981/. (Accessed 30 June 2017).

[15] Siranosian K. Many hospitals now safely reuse ‘single use’ medical devices. 2010. Available at http://amdr.org/2010/03/triplepundit-com-many-hospitals-now-safely-reuse-single-use-medical-devices/. (Accessed 29 June 2017).

[16] Hailey D, Jacobs PD, Ries NM, Polisena J (2008). Reuse of single use medical devices in Canada: clinical and economic outcomes, legal and ethical issues, and current hospital practice. Int J Technol Assess Health Care.

[17] Polisena J, Hailey D, Moulton K, Noorani HZ, Jocobs P, Ries N, Normandin S, Gardam M (2008). Reprocessing and reuse of single-use medical devices: a National Survey of Canadian acute-care hospitals. Infect Cont Hosp Ep.

[18] EMERGO. EUROPE – Overview of medical device industry and healthcare statistics. Available at https://www.emergobyul.com/resources/market-europe. (Accessed 30 June 2017).

[19] Ischinger TA, Neubauer G, Ujlaky R, Schätzl H, Bock M (2002). Wiederverwendung von medizinischen Einwegprodukten nach qualitätsgesicherter Wiederaufbereitung: ein Modell zur Kostendämpfung?. Z Kardiol.

[20] Mundo E. Material médico, se usa pero no se tira. 2005. Available at http://www.belt.es/noticias/2005/septiembre/21/mat_medico.asp. (Accessed 29 June 2017).

[21] Christensen M, Meyer M, Jensen OB (1999). Reuse of single-use sterile medical devices in Danish hospitals decreased after report discouraged it. Euro Surveill.

[22] Großkopf V, Jäkel C. Legal framework conditions for the reprocessing of medical devices. GMS Krankenhaushygiene Interdisziplinär 2008; 3: Doc24 (20080903). Available at https://www.egms.de/static/de/journals/dgkh/2008-3/dgkh000122.shtml. (Accessed 6 July 2017). PMC2831257 20204096

[23] Ulmer T. Reprocessing Medical Devices in Europe. 2007. Available at https://healthmanagement.org/c/hospital/issuearticle/reprocessing-medical-devices-in-europe. (Accessed 23 Apr 2019).

[24] Lane E. Reprocessing and reuse of single use medical devices: examination of ethical practice. Available at http://thisgreengrass.pbworks.com/w/file/fetch/50609886/emma%20lane%20position%20paper.pdf . (Accessed 12 July 2017).

[25] Koh A, Kawahara K (2005). Current practices and problems in the reuse of single-use devices in Japan. J Med Dent Sci.

[26] Jang Y-C, Lee C, Yoon O-S, Kim H (2006). Medical waste management in Korea. J Environ Manag.

[27] Hutin YJF, Hauri AM, Armstrong GL (2003). Use of injections in healthcare settings worldwide, 2000: literature review and regional estimates. BMJ.

[28] Janjua NZ, Akhtar S, Hutin YJF (2005). Injection use in two districts of Pakistan: implications for disease prevention. Int J Qual Health Care.

[29] Ahmad K (2004). Pakistan: a cirrhotic state?. Lancet.

[30] WHO. Single Use of Injection Devices. Patient Safety Solutions. 2007;1.

[31] Buchdid Amarante JM, Toscano CM, Pearson ML, Roth V, Jarvis WR, Levin AS (2008). Reprocessing and reuse of single-use medical devices used during hemodynamic procedures in Brazil: a widespread and largely overlooked problem. Infect Cont Hosp Ep.

[32] CFDA. Regulations for the Supervision and Management of Medical Devices. Available at http://www.nmpa.gov.cn/WS04/CL2185/300561.html. (Accessed 12 Sept 2017).

[33] Wu J, Zhou D (2011). Discussion on reprocessing expensive single use medical devices. Chinese Hospitals.

[34] The state health and family planning commission of the People’s Republic of China. Measures of the People’s Republic of China on the ethical review of Biomedical Research in People. Available at http://www.gov.cn/gongbao/content/2017/content_5227817.htm. (Accessed 18 Jan 2019).

